# A Deep Learning Decision Support Tool to Improve Risk Stratification and Reduce Unnecessary Biopsies in BI-RADS 4 Mammograms

**DOI:** 10.1148/ryai.220259

**Published:** 2023-08-09

**Authors:** Chika F. Ezeana, Tiancheng He, Tejal A. Patel, Virginia Kaklamani, Maryam Elmi, Erika Brigmon, Pamela M. Otto, Kenneth A. Kist, Heather Speck, Lin Wang, Joe Ensor, Ya-Chen T. Shih, Bumyang Kim, I-Wen Pan, Adam L. Cohen, Kristen Kelley, David Spak, Wei T. Yang, Jenny C. Chang, Stephen T. C. Wong

**Affiliations:** From the Department of Systems Medicine and Bioengineering, Houston Methodist Neal Cancer Center, Houston Methodist Hospital, Houston, Tex (C.F.E., T.H., L.W., S.T.C.W.); Houston Methodist Neal Cancer Center, Houston Methodist Hospital, Houston, Tex (J.E., J.C.C.); Departments of General Oncology (T.A.P.), Health Services Research (Y.C.T.S., B.K., I.W.P.), and Radiology (D.S., W.T.Y.), University of Texas MD Anderson Cancer Center, Houston, Tex; University of Texas Health Science Center, San Antonio, Tex (V.K., M.E., E.B., P.M.O., K.A.K.); University of the Incarnate Word School of Osteopathic Medicine, San Antonio, Tex (H.S.); Huntsman Cancer Institute, University of Utah, Salt Lake City, Utah (A.L.C., K.K.); and Department of Radiology, Houston Methodist Hospital, Weill Cornell Medicine, 6670 Bertner Ave, Houston, TX 77030 (S.T.C.W.).

**Keywords:** Mammography, Breast, Oncology, Biopsy/Needle Aspiration, Radiomics, Precision Mammography, AI-augmented Biopsy Decision Support Tool, Breast Cancer Risk Calculator, BI-RADS 4 Mammography Risk Stratification, Overbiopsy Reduction, Probability of Malignancy (POM) Assessment, Biopsy-based Positive Predictive Value (PPV3)

## Abstract

**Purpose:**

To evaluate the performance of a biopsy decision support algorithmic model, the intelligent-augmented breast cancer risk calculator (iBRISK), on a multicenter patient dataset.

**Materials and Methods:**

iBRISK was previously developed by applying deep learning to clinical risk factors and mammographic descriptors from 9700 patient records at the primary institution and validated using another 1078 patients. All patients were seen from March 2006 to December 2016. In this multicenter study, iBRISK was further assessed on an independent, retrospective dataset (January 2015–June 2019) from three major health care institutions in Texas, with Breast Imaging Reporting and Data System (BI-RADS) category 4 lesions. Data were dichotomized and trichotomized to measure precision in risk stratification and probability of malignancy (POM) estimation. iBRISK score was also evaluated as a continuous predictor of malignancy, and cost savings analysis was performed.

**Results:**

The iBRISK model's accuracy was 89.5%, area under the receiver operating characteristic curve (AUC) was 0.93 (95% CI: 0.92, 0.95), sensitivity was 100%, and specificity was 81%. A total of 4209 women (median age, 56 years [IQR, 45–65 years]) were included in the multicenter dataset. Only two of 1228 patients (0.16%) in the “low” POM group had malignant lesions, while in the “high” POM group, the malignancy rate was 85.9%. iBRISK score as a continuous predictor of malignancy yielded an AUC of 0.97 (95% CI: 0.97, 0.98). Estimated potential cost savings were more than $420 million.

**Conclusion:**

iBRISK demonstrated high sensitivity in the malignancy prediction of BI-RADS 4 lesions. iBRISK may safely obviate biopsies in up to 50% of patients in low or moderate POM groups and reduce biopsy-associated costs.

**Keywords:** Mammography, Breast, Oncology, Biopsy/Needle Aspiration, Radiomics, Precision Mammography, AI-augmented Biopsy Decision Support Tool, Breast Cancer Risk Calculator, BI-RADS 4 Mammography Risk Stratification, Overbiopsy Reduction, Probability of Malignancy (POM) Assessment, Biopsy-based Positive Predictive Value (PPV3)

*Supplemental material is available for this article.*

Published under a CC BY 4.0 license.

See also the commentary by McDonald and Conant in this issue.

## Graphical abstract

**Figure:**
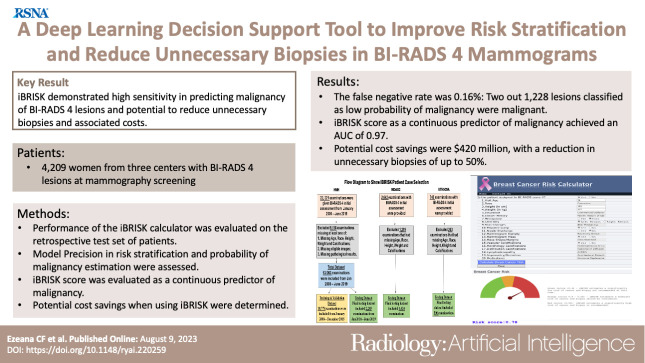

**Summary**

The intelligent-augmented breast cancer risk calculator (or, iBRISK) demonstrated potential to serve as an adjunct to Breast Imaging Reporting and Data System (BI-RADS) to improve risk stratification of BI-RADS category 4 lesions and reduce unnecessary biopsies in patients with lesions with low probability of malignancy.

**Key Points**

■ The intelligent-augmented breast cancer risk calculator (iBRISK) was developed to assess probability of malignancy of Breast Imaging Reporting and Data System (BI-RADS) category 4 lesions.
■ The iBRISK model achieved an accuracy of 89.5%, area under the receiver operating characteristic curve of 0.93 (95% CI: 0.92, 0.95), sensitivity of 100%, and specificity of 81%; only 0.16% of lesions determined to have a low probability of malignancy (POM) by the model were malignant, and lesions with high POM had a biopsy-proven predictive value of 85.9%.
■ A cost savings analysis demonstrated that iBRISK can reduce unnecessary biopsies of BI-RADS category 4 lesions by up to 50% in patients with lesions classified as low or moderate POM and can reduce financial costs.

## Introduction

Screening mammography is performed for early detection of breast cancer before clinically detectable signs of disease manifest (1–4). A major limitation of mammography is that patients with suspicious mammographic findings, such as Breast Imaging Reporting and Data System (BI-RADS) category 4 lesions, are recommended for biopsies that often yield benign outcomes. Of the more than 1 million breast biopsies performed annually in the United States, up to 75% have benign findings (5,6). Despite substantial research over the decades, tissue biopsy-proven positive predictive value (PPV3) (7) has not improved much (8). Thus, radiologists would benefit from tools or adjuncts to the BI-RADS system to more precisely assess breast cancer probability in women with BI-RADS category 4 lesions.

The American College of Radiology developed the BI‐RADS lexicon that standardizes mammographic reporting to facilitate cancer risk communication and biopsy recommendation (9). However, substantial inter- and intraobserver variability in the application of BI‐RADS remains, resulting in variation in biopsy rates across the United States (10,11) More importantly, established risk factors associated with breast cancer such as personal or family history of cancers, hormone replacement therapy, obesity, diabetes, hypertension, and so forth are not incorporated into the clinical decision model. The inclusion of these factors could contribute to a more robust and holistic assessment of a suspicious mammographic finding (Table S1) (12–14). The BI-RADS category 4 subgroup assigns wide variability in the probability of malignancy (POM), ranging from 2% to 95%. Biopsies of BI-RADS category 4 lesions serve as a quality metric and performance standard (15–18). False-positive mammograms are estimated to cost around $4 billion in the United States yearly (19).

A recent report based on the National Mammography Database subcategorized the majority of BI-RADS category 4 cases into BI-RADS category 4A (55.6%) and BI-RADS category 4B (31.8%), with associated low PPV3s of 7.6% and 22%, respectively (8). These findings indicate the opportunity for exploring, developing, and validating precision diagnostic models that can appropriately downgrade low and moderate suspicious assessments to nonactionable levels. Supplemental tools and algorithms would have less impact on BI-RADS category 4C lesions because they are fewer (12.6%) and of much higher PPV3 (69.3%).

Several models (20–25) have been developed previously, but they differ from our work in terms of scope, model predictors, and performance accuracy. Some earlier models incorporate all BI-RADS categories or focus on either screening or diagnostic data only. Other models are strictly limited to imaging data or integrate few clinical parameters and are often trained on public breast cancer screening datasets only.

We aim to evaluate the performance of our improved biopsy decision support algorithmic model, the intelligent-augmented breast cancer risk calculator (26) (iBRISK), on a large patient dataset in a multicenter study.

## Materials and Methods

### Source of Data

The institutional review boards of the participating institutions approved this study, which was performed in strict compliance with Health Insurance Portability and Accountability Act guidelines, and granted waivers of informed consent. We used retrospective, multi-institutional datasets to assess the performance of our previously developed and now improved iBRISK, a decision support tool that characterizes breast lesions classified as BI-RADS category 4 on mammograms and stratifies women according to POM (26). Data for this validation study include patient clinical data and mammography reports, which were consecutively drawn from the systemwide data warehouse of our institution, Houston Methodist Hospital (HMH) (27) (the same data source for model development and improvement [March 2006–December 2016]). Data were also consecutively curated from the electronic medical records from MD Anderson Cancer Center (MDACC) (March 2016–September 2018) and the University of Texas Health Science Center San Antonio (UTHSCSA) (January 2015–June 2019). Our study evaluated only patients with BI-RADS category 4 lesions with mammographic abnormalities. To limit the number of input variables and keep the model user-friendly, mammography alone was used. Also, the final features of the iBRISK model were purely from mammographic descriptors and clinical factors.

### Patients

**Figure 1:**
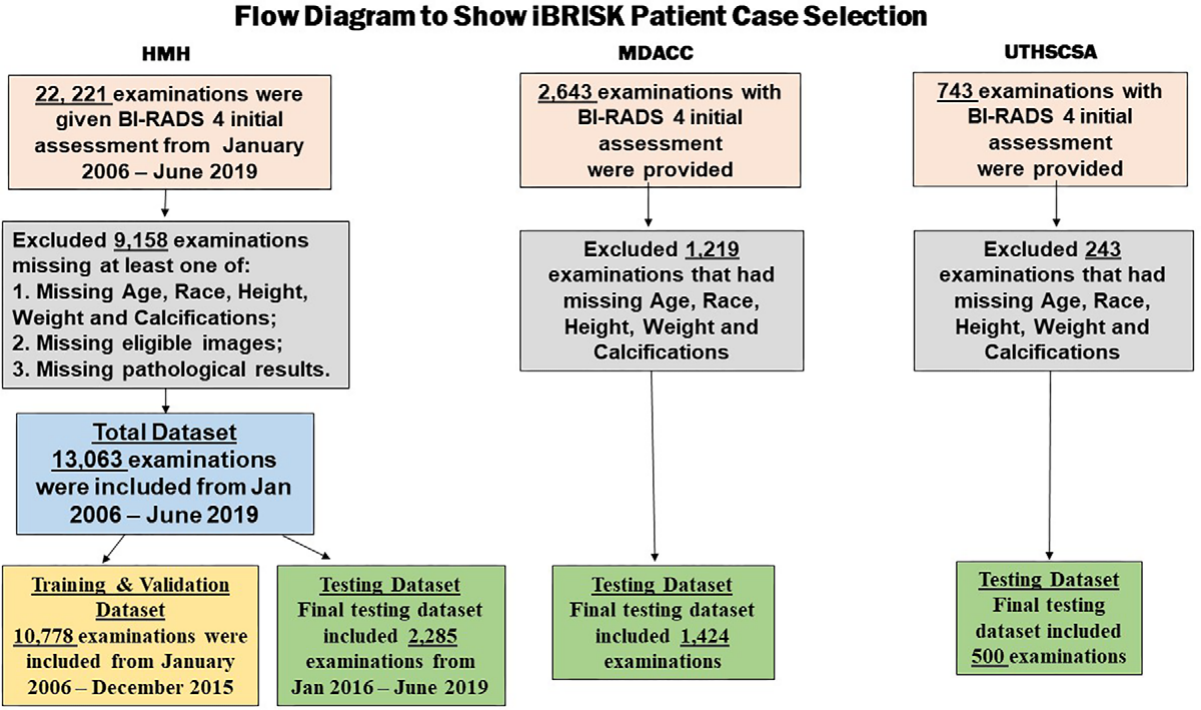
Flow diagram shows patient case selection for refined model training, validation, and multicenter testing. BI-RADS = Breast Imaging Reporting and Data System, iBRISK = intelligent-augmented breast cancer risk calculator.

The study included patients with lesions categorized as BI-RADS 4 at diagnostic mammography, including recalls from screening, who were seen consecutively in diverse clinical settings. Minimum inclusion criteria were data on age, height, weight, and calcification details at imaging, as well as a biopsy performed within 3 months after mammography. Patients with lesions classified into other BI-RADS categories or missing the aforementioned data were excluded (Fig 1). The study used only retrospective patient data; there was no direct patient contact, and patients did not receive any treatments.

### Outcome

Each patient in the test dataset was evaluated using iBRISK by inputting the individual's set of 20 variables (Table S1) comprising the model, which were derived from clinical factors and mammographic descriptors (Table S1). Please see Appendix S1 for details on the iBRISK model (26), its improvement and updating, comparison with other models, and predictors. The calculator provided a POM score between 0 and 1, as well as biopsy decision-making support recommendations based on these scores. Evaluations were performed while blinded to patients’ pathologic results. Afterward, model results were compared with biopsy outcomes, which served as ground truth.

### Missing Data

The validation data of HMH and MDACC were complete datasets without missing values. UTHSCSA, however, had 1.34% missing data. Thus, of 20 features each for all 500 cases (ie, 10 000 observations from this site), 134 observations were missing. We used one-hot encoding (28,29) to vectorize the input parameters, and after the parameters were vectorized, the binary values in the missing data became all zeroes (0,0), compared with (0,1) for “yes” and (1,0) for “no”. Interestingly, continuous input variables like age, height, and weight were and would always be available in real-time workflow.

### Statistical Analysis

An iBRISK score was derived for each patient (*n* = 4209). A single continuous predictor logistic regression model was fitted to the data to test the ability of the iBRISK scores to predict pathologic findings. The iBRISK scores were trichotomized into “low,” “moderate,” or “high” POM (cutoff points were determined based on the dynamics of our training data and model settings) and correlated with the pathologic findings (a dichotomous categorical factor: malignant or benign), which served as ground truth in a χ^2^ test. Additionally, the model scores were dichotomized into low versus “not low” or “not high” versus high and then correlated with the same χ^2^ analysis of the pathologic findings (benign or malignant). In either case, in the χ^2^ analysis, we correlated the iBRISK trichotomized or dichotomized predictor with the pathologic finding. Receiver operating characteristic (ROC) curve analysis for all patients in the test set, as well as subdivision by race classifications in the data warehouse and electronic medical records, was performed, and the area under the ROC curve (AUC) was calculated. An assessment of the impact of missing variables on model accuracy was performed using data from MDACC (1424 patients); that is, we simulated states of missing features by progressively removing one feature at a time and assessing the impact on model accuracy and stability. We estimated possible iBRISK-assisted biopsy avoidance, and potential cost savings were calculated in a cost analysis.

To assess the importance of each of the 20 factors in the model, 20 unique sets of scores were derived in which each set of scores represented the effect of removing a different factor, that is, without imputation of said factor's value. Logistic regression was used to derive AUCs for each of the 20 sets of scores as a marker of model performance. Differences in performance (as measured by the areas under the empirical ROC curves) between the full and factor-restricted models were assessed using the method of DeLong et al (30). Decreases in AUC, along with their associated 95% Wald CIs, between the full model and each of the cluster-restricted models were used to measure cluster effect on model performance. The significance of POM differences between groups was determined using χ^2^ tests. A *P* value of less than .05 was considered statistically significant. All analyses were conducted using SAS 9.4 software (SAS Institute) (31).

## Results

### Patient Characteristics

The testing dataset (4209) comprised 2285 of 3887 (58.8%) patients from HMH, 1424 of 2643 (53.9%) from MDACC, and 500 of 743 (67.3%) from UTHSCSA (Fig 1). Median and IQR values for the multicenter validation dataset (*n* = 4209) were as follows: age, 56 years (IQR, 45–65 years); height, 162.6 cm (IQR, 153.8–167.9 cm); weight, 85.73 kg (IQR, 59.3–87.1 kg); and body mass index, 32.08 (IQR, 23–34). Non-Hispanic White individuals (2504 of 4209 [59.49%]) and commercial insurance and self-paying patients (3006 of 4209 [71.42%]) were the majority for race and insurance status, respectively. Descriptive statistics of other demographic predictors can be found in Tables 1 and 2.

**Table 1:**
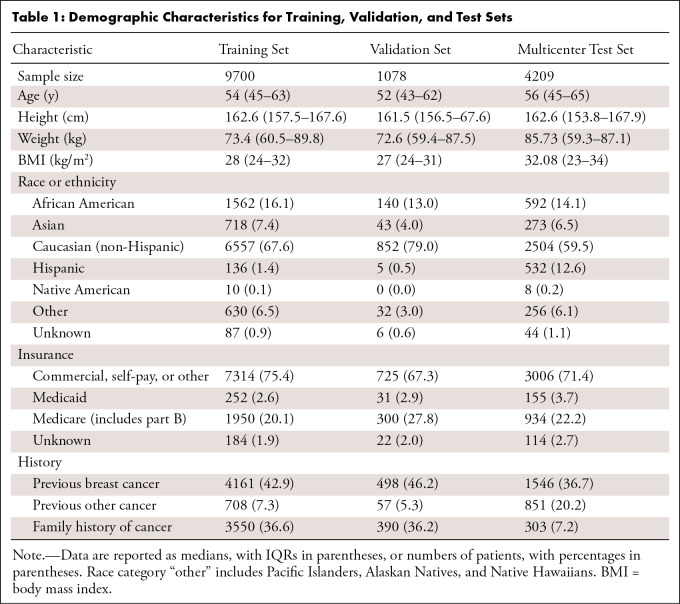
Demographic Characteristics for Training, Validation, and Test Sets

**Table 2:**
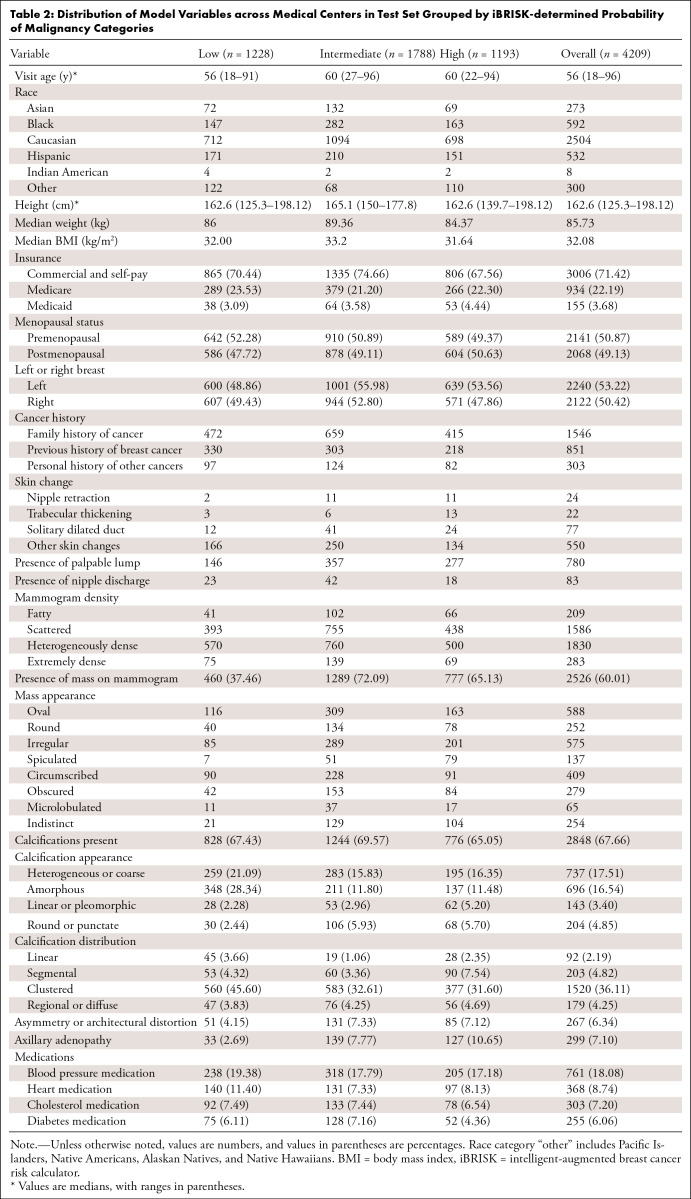
Distribution of Model Variables across Medical Centers in Test Set Grouped by iBRISK-determined Probability of Malignancy Categories

### Model Performance

**Figure 2:**
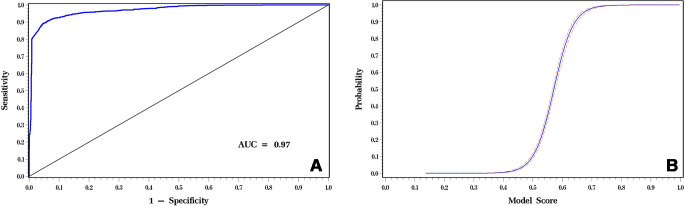
**(A)** Receiver operating characteristic curve of iBRISK score as a continuous estimate of the probability of breast lesion malignancy. **(B)** Graph shows probability of malignancy by model score (logistic estimate with confidence limits). AUC = area under the receiver operating characteristic curve, iBRISK = intelligent-augmented breast cancer risk calculator.

***Score as a continuous predictor.—*** Logistic regression was fitted to the data, assuming model score as a continuous predictor of malignancy, resulting in an AUC of 0.97 (95% CI: 0.96, 0.98) (Fig 2A). Figure 2B shows the graph for the logistic estimate. The model suggests the POM is zero for scores less than 0.4 and extremely high for scores above 0.7, as indicated by the trichotomized data. Figure S1 outlines the scores as a continuous predictor according to race and ethnicity distribution.

***Trichotomized into low, moderate, and high POM.—*** iBRISK designated patients as having low POM for scores of less than 0.4, moderate POM for scores between 0.4 and 0.55, and high POM for scores greater than 0.55. Overall, 29.2% (1228 of 4209) of patients in the multicenter validation dataset had low POM, 42.8% (1788 of 4209) had moderate POM, and 28.1% (1193 of 4209) had high POM. While the distribution of patients in the three POM categories was significantly different between institutions (Fig S2), the calculator performed equally at all three sites in terms of sensitivity, accuracy, and when dichotomized as described below.

**Figure 3:**
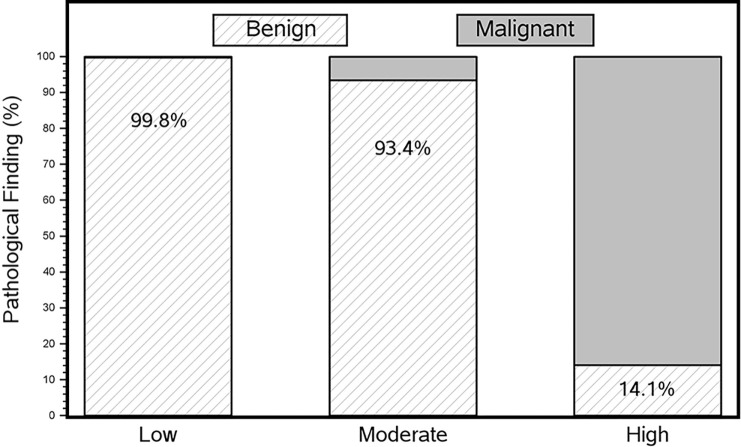
Percentage of benign and malignant pathologic findings after biopsy of breast lesions according to iBRISK probability of malignancy level. iBRISK = intelligent-augmented breast cancer risk calculator.

The proportion of benign lesions within these 4209 patients was significantly different between the POM groups (*P* < .001). When the model predicted a low POM, the likelihood of a benign biopsy finding was 99.8% (Fig 3), with a false-negative rate (FNR) of 0.16% (two of 1228 were malignant). Most patients in the moderate POM category also had benign biopsy findings (93.4%, 1670 of 1788), with a slightly higher malignancy rate of 6.6% (118 of 1788). The high POM group had a malignancy rate of 85.9% (1025 of 1193). The calculator designated only 14.1% (168 of 1193) of benign biopsy findings as high POM (false-positive rate [FPR]) (Table 3, Fig 3).

**Table 3:**
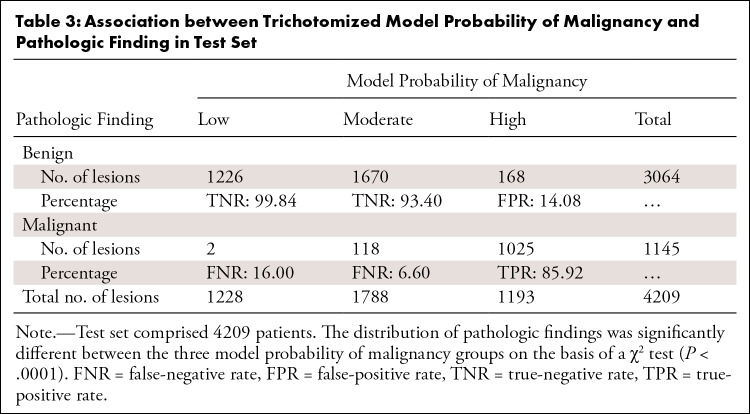
Association between Trichotomized Model Probability of Malignancy and Pathologic Finding in Test Set

***Dichotomized into low versus not low POM.—*** Model scores were dichotomized into low versus not low POM following the clinical decision process in the 4209 patients. The proportion of benign lesions was significantly different between the two risk groups (*P* < .001). As previously mentioned, there were 1228 patients in the low POM group (FNR, 0.16%). There were 1838 patients in the not low POM group who would have undergone biopsy with benign results, with an FPR of 61.66%. Model sensitivity was 99.83% (ie, the model would detect malignant lesions 99.83% of the time) (Table 4). Performance metrics between groups per institution are shown in Table S2.

**Table 4:**
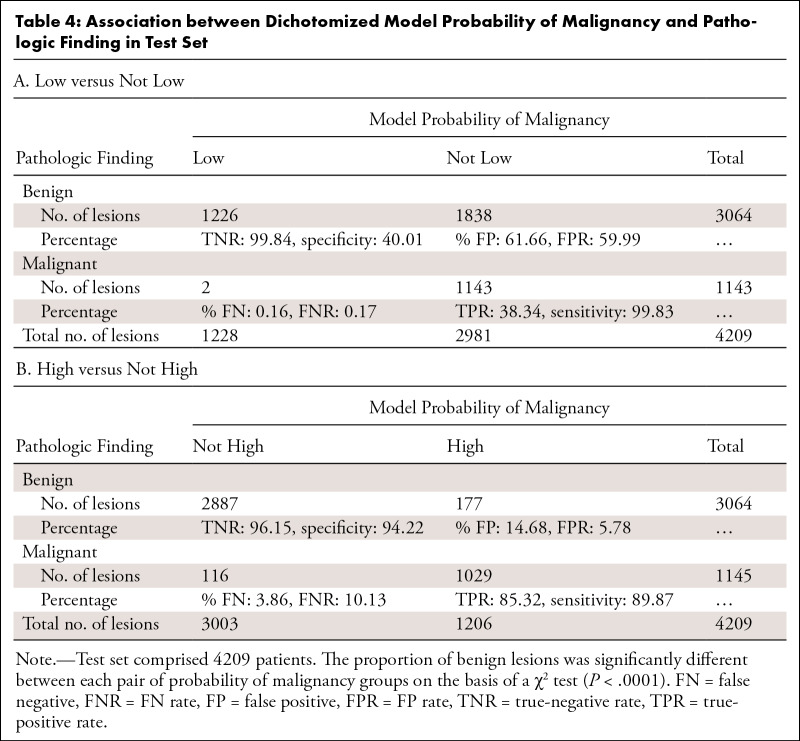
Association between Dichotomized Model Probability of Malignancy and Pathologic Finding in Test Set

***Dichotomized into high versus not high POM.—*** When model scores were dichotomized into high versus not high POM, the proportion of benign lesions was significantly different between the two risk groups (*P* < .001)*.* Among the lesions categorized as not high POM, 116 were malignant (FNR, 3.86%), while 177 biopsies would have been conducted among patients with benign lesions (FPR, 14.68%). The model achieved 89.87% sensitivity, 94.22% specificity, and an AUC of 0.92 (Table 4; per institution, Table S2). Table S3 shows the percentage of benign and malignant biopsy findings and the FNRs and FPRs after model categorization of lesions as low, moderate, or high POM.

**Figure 4:**
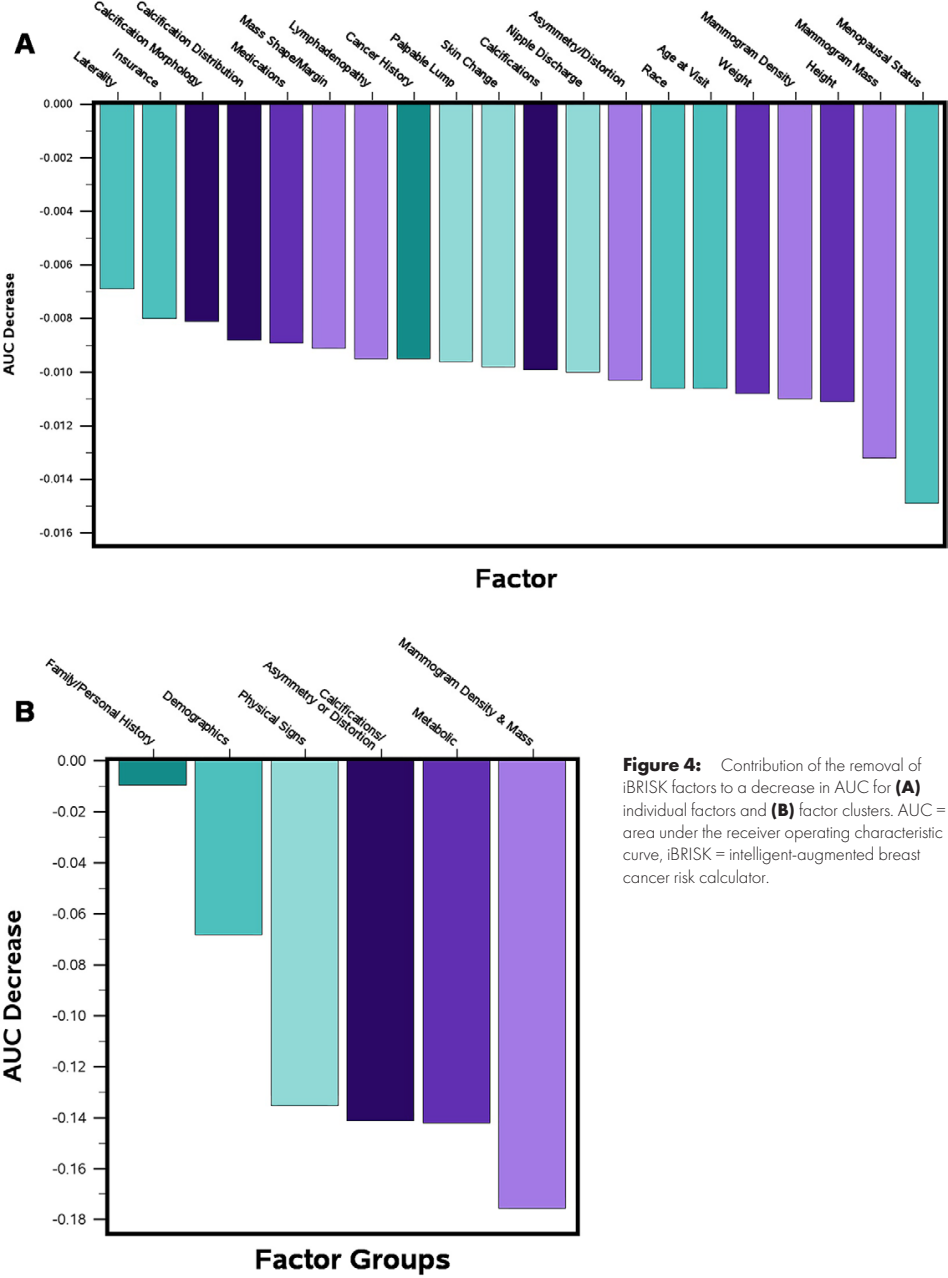
Contribution of the removal of iBRISK factors to a decrease in AUC for **(A)** individual factors and **(B)** factor clusters. AUC = area under the receiver operating characteristic curve, iBRISK = intelligent-augmented breast cancer risk calculator.

***Contribution of factors in iBRISK, individually and in clusters.—*** The contribution of each of the 20 factors in iBRISK was calculated using simple logistic regression to estimate the AUC after removing each factor from the model. Each factor removal resulted in a small but statistically significant decrease in performance, reflecting its relative contribution. Menopausal status and mammographic mass had the largest contributions according to decrease in AUC (Fig 4A, Table 5). We grouped the final 20 factors in the model into the following six clusters: *(a)* demographics (age, race, menopausal status, and laterality), *(b)* metabolic factors (height, weight, insurance, and medications, including hormone replacement therapy), *(c)* history (personal history of breast or other cancers, family history of breast or other cancers), *(d)* physical signs (skin changes, nipple discharge, palpable lump, and lymphadenopathy), *(e)* mammographic density and mammographic mass, and *(f)* mammographic calcification features (vascular calcification, calcification morphology, and calcification distribution) and asymmetry and architectural distortion. Mammographic mass, metabolic factors, and mammographic calcification features showed the highest contributions (Fig 4B, Table 5).

**Table 5:**
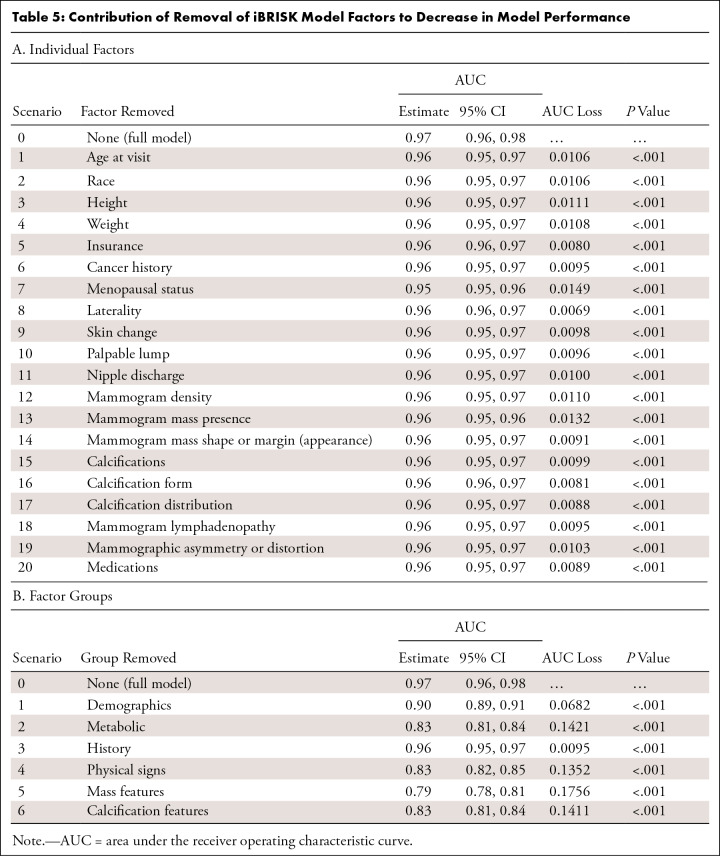
Contribution of Removal of iBRISK Model Factors to Decrease in Model Performance

### Missing Feature Analysis

**Figure 5:**
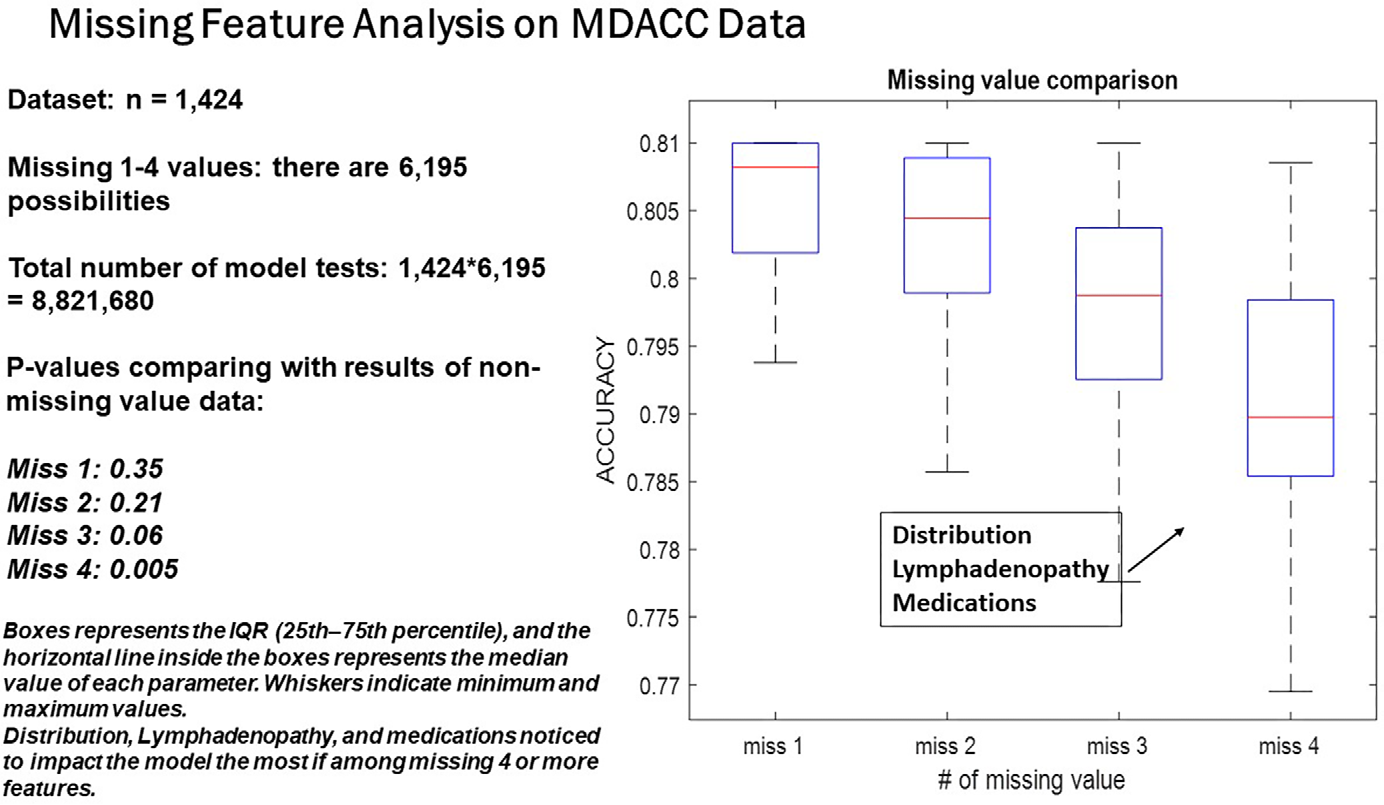
Missing feature analysis of MDACC dataset shows a slight drop in iBRISK accuracy with each additional missing feature. iBRISK = intelligent-augmented breast cancer risk calculator, MDACC = MD Anderson Cancer Center.

Using MDACC data (*n* = 1424) to assess the impact of missing features on the accuracy and stability of the iBRISK model, we observed progressively slighter declines with each additional missing feature and a statistically significant level drop in accuracy when the fourth feature was removed. Thus, the model can tolerate up to three missing features while retaining robustness and confidence in the accuracy of results generated (Fig 5).

### Estimated Annual Cost Savings

Table S4 shows the median projected cost (based on the Medicare reimbursement rate) of biopsy ($380) and the average cost for each type of biopsy. The most common type of biopsy among MDACC patients was stereotactic biopsy (68%). The cost of biopsy ranged from $321 (stereotactic biopsy) to almost $3600 (mammography-guided surgical biopsy). Table S5 provides the information used to derive our estimate of cost savings as a result of triaging patients classified as low risk by iBRISK to not undergo biopsy. As shown, biopsy can potentially be avoided for approximately 390 000 women, with cost savings of more than $420 million.

## Discussion

We improved iBRISK and evaluated the model by using a retrospective multi-institutional dataset made up of patients from MDACC, UTHSCSA, and HMH. iBRISK demonstrated high sensitivity and specificity for the prediction of POM, resulting in improved risk stratification of BI-RADS category 4 lesions, such that only 0.16% (two of 1228) of lesions classified as low POM in women assessed by iBRISK were malignant, and PPV3 among the high POM group was 85.9% (1025 of 1193), which is close to that of the BI-RADS 5 risk category (80.3%–97.9%) and outperforms radiologists’ BI-RADS 4 categorization accuracy (20,32,33). Thus, iBRISK can potentially obviate up to 50% of biopsies in patients with BI-RADS 4 mammograms.

The iBRISK calculator can assist physicians, primarily radiologists, in triaging patients to low POM groups to avoid biopsies of benign lesions, while high-risk groups can be treated as patients with BI-RADS category 5 lesions. A more precise stratification system that considers vital patient characteristics in addition to abnormal, suspicious imaging features is needed to enhance POM estimation to guide the safe management of such mammographic findings, prevent overbiopsy and associated costs, and reduce patient emotional distress (34,35). The goal is not to replace or modify the BI-RADS standards but to improve precision in predicting the malignancy of category 4 lesions, which are currently overbiopsied, when iBRISK is used alongside the BI-RADS system.

While various models have been proposed and several studies performed (20,21,24,25), a safe, pragmatic, and effective system that addresses these concerns has not been reported. A 2015 study included the Gail model, body mass index, and genetic marker information for breast cancer risk estimation in women with suspicious findings on BI-RADS 4 mammograms (21). Similar to our current study, this study considered clinical factors, albeit in a more limited fashion, but did not improve POM estimation precision within the BI-RADS 4 category. A 2019 study proposed a combined machine and deep learning approach applied to digital mammograms and electronic health records to identify false-negative findings in BI-RADS categories 1, 2, and 3 (24). The algorithm identified 34 of 71 (48%) of such findings on mammograms. Another study in 2021 developed a deep learning fusion network model using mammography imaging biomarkers and clinical features of BI-RADS category 3, 4, and 5 lesions to predict malignancy (25). However, their test cohort was relatively small (internal test cohort, 244 patients; external test cohort, 100 patients). Most current published works do not compare results with the BI-RADS guidelines, address the issue of precision POM estimation of BI-RADS category 4 mammogram suspicious findings, or address the issue of overbiopsy, and do not involve multiple nonimaging parameters. Further, current published artificial intelligence models for cancer probability estimation were developed using clinical images from a public breast cancer screening dataset (22,23,36) and do not incorporate the above parameters.

Because of variability in malignancy rates associated with BI-RADS category 4, the BI-RADS fifth edition proposes the following subcategories for likelihood of malignancy: 4A (2%–10%), 4B (11%–50%), and 4C (50%–95%). Reported PPV3 for BI-RADS category 4 ranged from 15% to 30% (37–40) and was recently reported as 21.1% (4A: 7.6%, 4B: 22.2%, 4C: 69.3%) (8) in the United States and between 21% and 27.1% in other countries (32,41). Subcategorization of BI-RADS 4 is not widely adopted, as the malignancy rate in the 4A (low risk) category is up to 10% (20). Our study demonstrated that iBRISK outperforms BI-RADS subcategory recommendations, with an FNR of less than 1% in the low POM category. There were malignancy rates of 6.6% in the iBRISK moderate POM group, which is still lower than the published BI-RADS category 4A range, and 85.9% in the high POM group, close to the BI-RADS 5 category. Also, a recent published study found that essentially, in the BI-RADS 4 category, digital breast tomosynthesis had no comparative advantage over digital mammography in terms of PPV3 and cancer detection rate, which could answer questions on the impact of the broad implementation of digital breast tomosynthesis on precision (42).

Our study had certain limitations. First, the iBRISK model was built, refined, and internally validated with patient data from a major health system in the greater Houston area, and this reported multi-institutional testing is largely restricted to data from three leading hospitals in Texas. A larger multicenter study involving other states is being planned to further assess the model's performance in more diverse patient populations and breast imaging practices. Second, the study required complete retrospective data curation to incorporate both mammographic and patient risk factors not readily considered in mammography reports, resulting in 53.9%–67.3% of patients from the initial datasets at the three participating sites to be included in the final analysis. However, the model performed well across the sites, suggesting model robustness to external data. Third, 85% of the 20 model variables were needed for robust POM scoring using iBRISK, with the calculator tolerating a maximum of three missing features. The slight accuracy decreases with each additional missing feature using the MDACC dataset emphasize the requirement of precise data curation and annotation for the optimal function of this tool. Fourth, iBRISK substitutes missing data with a default (unknown) or average number value, thus affecting its accuracy. However, demographic and specific information on mammographic calcifications would be available when used in real time. The lack of consistent reporting on mammographic calcification form and calcification distribution underscores the urgent need for consistent structured breast imaging reporting systems that optimize data acquisition, archiving, retrieval, and extraction. The envisaged clinical workflow is an online iBRISK calculator where these 20 features including demographics, history, and mammographic features would be inputted after a mammography examination with a BI-RADS category 4 designation by the radiologist or other providers and the risk score generated. Fifth, findings of previous scans have not been included because of availability constraints, though it can be argued that progression of calcifications and lesions over time is important. Extending our analysis beyond lesion classification as benign versus malignant to clinical outcomes, histology of cancer types, and aggressive versus less aggressive tumors was beyond the scope of this study and should be investigated in future studies. Sixth, most biopsies evaluated in the cost savings analysis were stereotactic biopsies. While other biopsies were performed at this site, the data retrieval and study period occurred during the migration of the electronic medical record system to a new platform; therefore, successful data curation with accurate clinical information of the other biopsy types could not be performed. Of note, stereotactic biopsies are much cheaper. Last, iBRISK POM assessment serves as an adjunct to breast imaging for clinical providers and patients in biopsy decision-making and thus is not a definitive diagnostic tool.

In summary, our study demonstrates that iBRISK can effectively aid in risk stratification of BI-RADS category 4 lesions and reduce overbiopsy of these lesions. Ultimately, the iBRISK calculator will be published as an online interface and made open access, noncommercial, and accessible by health systems and centers worldwide. Future studies aim to improve the model further, particularly by including more granular data and other BI-RADS categories.

**Figure 6:**
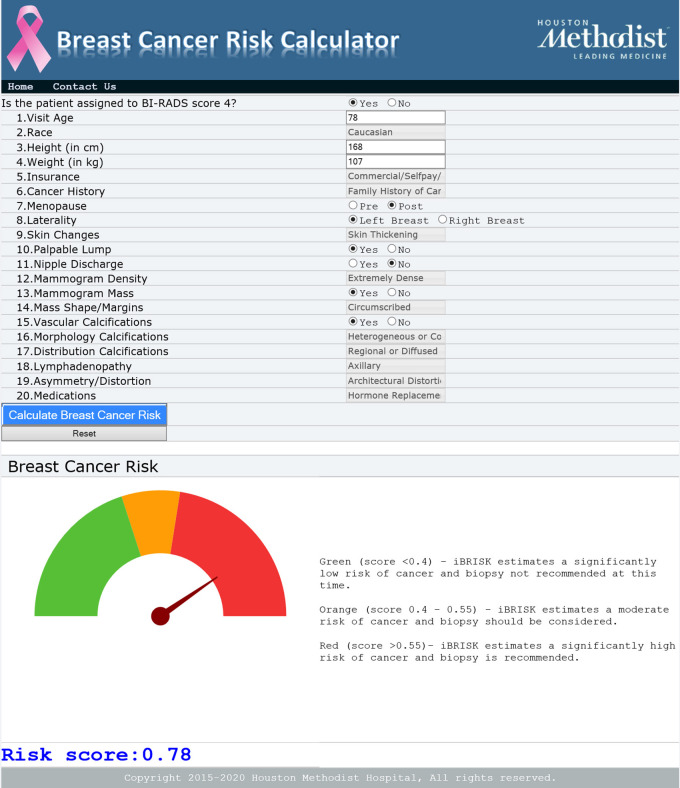
Online iBRISK interface showing 20 fields. iBRISK = intelligent-augmented breast cancer risk calculator.
